# Physical growth and neurodevelopment during the first year of life: a cohort study of the Japan Environment and Children’s Study

**DOI:** 10.1186/s12887-021-02815-9

**Published:** 2021-08-25

**Authors:** Masafumi Sanefuji, Yuri Sonoda, Yoshiya Ito, Masanobu Ogawa, Vlad Tocan, Hirosuke Inoue, Masayuki Ochiai, Masayuki Shimono, Reiko Suga, Ayako Senju, Satoshi Honjo, Koichi Kusuhara, Shouichi Ohga, Japan Environment and Children’s Study Group, Michihiro Kamijima, Shin Yamazaki, Yukihiro Ohya, Reiko Kishi, Nobuo Yaegashi, Koichi Hashimoto, Chisato Mori, Shuichi Ito, Zentaro Yamagata, Hidekuni Inadera, Takeo Nakayama, Hiroyasu Iso, Masayuki Shima, Youichi Kurozawa, Narufumi Suganuma, Koichi Kusuhara, Takahiko Katoh

**Affiliations:** 1 Research Center for Environment and Developmental Medical Sciences, Kyushu University, Fukuoka, Japan; 2 Department of Pediatrics, Graduate School of Medical Sciences, Kyushu University, Fukuoka, Japan; 3 Japanese Red Cross Hokkaido College of Nursing, Kitami, Japan; 4 Department of Pediatrics, University of Occupational and Environmental Health, Kitakyushu, Japan; 5 Regional Center for Japan Environment and Children’s Study, University of Occupational and Environmental Health, Kitakyushu, Japan; 6 Department of Pediatrics, National Hospital Organization Fukuoka National Hospital, Fukuoka, Japan

**Keywords:** Conditional growth modeling, Infant, Neurodevelopment, Physical growth

## Abstract

**Background:**

The association between a slower physical growth and poorer neurodevelopment has been established in infants born preterm or small for gestational age. However, this association is inconsistent in term-born infants, and detailed investigations in infancy, when intervention is most beneficial for improving outcomes, are lacking. We therefore examined this association separately by sex during the first year of life in term-born infants.

**Methods:**

Using data collected until children reached 12 months old in an ongoing prospective cohort of the Japan Environment and Children’s Study, we analyzed 44,264 boys and 42,541 girls with singleton term-birth. The exposure variables were conditional variables that disentangle linear growth from weight gain relative to linear growth, calculated from the length and weight at birth and 4, 7 and 10 months old. Neurodevelopmental delay was identified using the Japanese-translated version of Ages & Stages Questionnaires, third edition.

**Results:**

A reduced risk of neurodevelopmental delay at 6 months old was observed in children with a higher birth weight (adjusted relative risks [aRRs]: 0.91 and 0.93, 95 % confidence intervals [95 % CIs]: 0.87–0.96 and 0.88–0.98 in boys and girls, respectively) and increased linear growth between 0 and 4 months old (aRRs: 0.85 and 0.87, 95 % CIs: 0.82–0.88 and 0.83–0.91 in boys and girls, respectively). A reduced risk at 12 months was found in children with an increased linear growth between 0 and 4 months (aRRs: 0.92 and 0.90, 95 % CIs: 0.87–0.98 and 0.84–0.96 in boys and girls, respectively), boys with an increased relative weight gain between 0 and 4 months (aRR: 0.90, 95 % CI: 0.84–0.97), and girls with a higher birth weight (aRR: 0.89, 95 % CI: 0.83–0.96).

**Conclusions:**

These results suggest that a slow physical growth by four months old may be a predictor of neurodevelopmental delay during infancy.

**Supplementary Information:**

The online version contains supplementary material available at 10.1186/s12887-021-02815-9.

## Background

Prenatal and postnatal growth of the body has been linked to later neurocognitive development. Growth restriction *in utero* is suggested to occur in infants born small for gestational age while poor growth from birth to term-equivalent age is often observed in infants born preterm. Such growth-faltering infants are at high risk of poor neurodevelopmental outcomes later in life [1, 2]. In these infants, a lack of catch-up growth is an important indicator of a poor neurodevelopment [3–5]. The association between physical growth and neurodevelopment has also been examined in general populations consisting mostly of term-born infants, but the findings are less consistent in such settings. Indeed, while several studies with limited sample sizes have shown positive results [6–14], others have reported no association [15, 16]. Furthermore, as outcomes, a majority of these studies investigated the neurocognitive function during preschool through adulthood [6–10, 13–16] with only a few examining those in infancy [8, 11, 12], during which intervention has the potential to improve the outcomes of both the parents and their children [17, 18].

Most of these previous studies have separately assessed anthropometric gains in either length/height or weight between two age points (e.g. birth to six months old). As the length/height and weight are strongly correlated within an individual, assessing these indices *per se* hampers the determination of which component is more influential. To dissociate the effects of length/height growth from those of weight growth, Adair et al. and Horta et al. used a sophisticated method of ‘conditional growth modeling’ [19, 20]. This modeling determines conditional variables by regressing the current size (i.e. length or weight) against all previous sizes [21, 22]. With respect to each period, the variables comprise ‘linear growth’ and ‘weight gain relative to linear growth’, which represent length/height change and weight change separated from change in length/height, respectively. Their studies demonstrated a closer relationship of school achievement and intelligence with linear growth than with relative weight again, noting some differences in patterns between sexes.

Using a conditional growth model with a nationwide prospective cohort from the Japan Environment and Children’s Study (JECS) that includes over 100,000 children, the present study examined the relationship between physical growth and neurodevelopment separately by sex during the first year of life in term-born infants. To collect neurodevelopmental information from such a large number of children, we used a parent-reported screening tool rather than individualized face-to-face tests. During this period, neurodevelopment is difficult to assess precisely, and important changes can manifest in the individual developmental trajectory. However, a detailed investigation of this relationship during infancy is extremely important for detecting neurodevelopmental delay for early intervention.

## Methods

### Design

The JECS is a nationwide, multicenter, prospective birth cohort study funded by the Ministry of Environment, Japan. The study design details have been described elsewhere [23, 24]. In brief, pregnant participants were registered between January 2011 and March 2014 in 15 regional centers covering a wide geographical area in Japan. During pregnancy, data were obtained during the first and second/third trimesters using self-administered questionnaires. Detailed information regarding the mother and child was obtained from medical records transcripts during the first trimester, at the time of delivery, and when the child was 1 month old. After delivery, data were collected at one and six months old and every six months until the child was six years old, then twice a year thereafter *via* self-reported questionnaires completed by the parents.

The JECS protocol was reviewed and approved by the Ministry of Environment’s Institutional Review Board for Epidemiological Studies and by the ethics committees of all participating institutions (#15000141). The ethical approval for this study was an extension of the ethical approval for the JECS protocol. Written informed consent was obtained from all parents.

### Participants

**Fig. 1:**
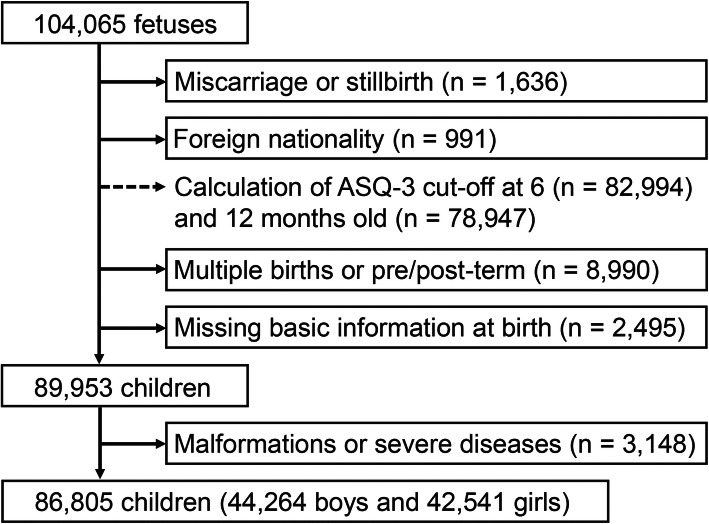
Flowchart of participant selection

In this study, we used the fixed dataset “jecs-an-20180131” released in March 2018. The dataset includes all available data of 104,065 fetuses, linked to their mothers’ data, collected until children were 12 months old. As subjects, we selected 89,953 children with live singleton term birth (≥ 37 and < 42 gestational weeks) who had parents of Japanese nationality and complete basic information on mother’s parity, child’s sex, length and weight at birth (Fig. 1). After excluding 3,148 children who had malformation/severe diseases, we eventually analyzed 86,805 children, including 44,264 boys and 42,541 girls.

### Exposure

The exposure variable was body growth from conception to 10 months old. Length and weight at birth, which can be viewed as growth from conception (length and weight = 0) to birth, were obtained from medical records. Subsequent anthropometric data were all derived from the personal health record of the child. Nearly all Japanese children receive a public health checkup at four months old. Their length and weight were thus directly measured by a public health nurse or a hospital nurse and officially documented in the record. They also receive similar checkups at 7 or 10 months old, occasionally at both 7 and 10 months old, depending on residency. Parents were asked to transcribe these anthropometric data at the 4-month checkup in the 6-month questionnaire and those at the 7- and 10-month checkups in the 12-month questionnaire. Because the data were not measured at exactly 4, 7 and 10 months, the values were extrapolated from the anthropometric data at birth and those at the 4-, 7- and 10-month checkups [25]. The extrapolated data were referenced to the Japanese growth standards separately by sex and then converted into z-scores (i.e. length-for-age, weight-for-age) [26, 27]. In the standards, the mother’s parity and child’s gestational age are additionally taken into consideration when calculating z-scores at birth.

These z-scored length and weight values at different ages were processed using conditional growth modeling, as in the studies conducted by Adair et al. and Horta et al. [19, 20] but additionally including birth length in our study. Conditional variables were obtained by regressing the current size (i.e. length or weight) against all previous sizes [21, 22]. At each time point, a conditional variable represents physical growth during a time interval, and a positive value represents a linear growth or relative weight gain faster than predicted in that period. For example, the conditional length at seven months old indicates the linear growth from four to seven months old. To estimate the conditional length, the current length was regressed against all previous lengths and weights. The conditional length at seven months old was derived by regressing the length at seven months old against the length and weight at four months old and birth. To estimate the conditional relative weight, the current weight was regressed against the current length and all previous lengths and weights. For instance, the conditional relative weight at seven months old was calculated by regressing the weight at seven months old against the length at seven months old and the length and weight at four months old and birth. Note that the length and weight at birth *per se* were treated as conditional variables at birth, as they had no previous anthropometric data to be regressed against. Consequently, our conditional variables included length and weight at birth and conditional length and relative weight at 4, 7 and/or 10 months old.

### Outcomes

The outcome measure was neurodevelopmental delay at 6 and 12 months old using the Japanese-translated version of the Ages & Stages Questionnaires: third edition (ASQ-3). This version was prepared through a back-translation procedure and was approved by the publisher of the original English version [28]. The ASQ-3 can identify infants or young children who require further neurodevelopmental assessments to determine their eligibility for early intervention. The findings of the questionnaire basically agree with those of professionally administered developmental batteries [29, 30]. It has been used in clinical and research settings and translated into several languages [31–33].

The ASQ-3 assesses five developmental domains: Communication, Gross Motor, Fine Motor, Problem Solving, and Personal-Social domains. For each domain, six skills are described to which parents answer “yes,” “sometimes,” or “not yet,” depending on whether or not their child has demonstrated the described skill. The responses are converted to points, with “yes” receiving 10 points; “sometimes”, 5 points; and “not yet”, 0 points. The child’s score for each developmental domain is the sum of all points received for the items under that domain and ranges from 0 to 60 points. The cut-off score for each domain is defined as two standard deviations below the mean score of large, standardized samples in the United States of America. A child is defined as having a neurodevelopmental delay if a score is at or below the cut-off level in any developmental domain. Although preliminary cut-off scores of the Japanese translation were recently proposed [34], these were not recommended to be used with confidence before 24 months old because of very limited sample sizes. Therefore, the cut-off scores were determined using the same methodologies as the original version, based on available data at 6 (n = 82,994) and 12 months old (n = 78,947) (Fig. 1), which represent the general Japanese population.

### Statistical analyses

To examine which component and periods of physical growth are specifically associated with infant neurodevelopment, we conducted multivariable regression analyses using conditional variables at different ages. Because these variables are not correlated with each other, the variables and covariates were included together in the same model without concerns about collinearity [20]. The covariates were (i) mother’s age; (ii) maternal smoking during pregnancy, as recorded in the first trimester; (iii) maternal and (iv) paternal education level (junior high school, high school, and university or graduate school); (v) annual family income (< 4 000 000, 4 000 000–5 999 999, ≥ 6 000 000 JPY); (vi) home speech stimulation at 1 month old (whether a mother did or did not talk to her baby habitually). The ‘home speech stimulation’ covariate was used instead of the Home Observation for Measurement of the Environment scale [35], which is not employed in the JECS.

All analyses were performed using the R software program, version 4.0.3 (The R Foundation, Vienna, Austria). Quasi-Poisson regression models were used to estimate adjusted relative risks (aRRs) with 95 % confidence intervals (CIs).

## Results

Among the 44,264 boys and 42,541 girls eligible for the analysis, anthropometric data were available at 4 months of age in 37,136 (83.9 %) boys and 35,732 (84.0 %) girls, at 7 months in 16,231 (36.7 %) boys and 15,758 (37.0 %) girls, and at 10 months in 25,616 (57.9 %) boys and 24,570 (57.8 %) girls, respectively. Ultimately, 10,016 (22.6 %) boys and 9,801 (23.0 %) girls had complete data at all timepoints of birth, 4, 7 and 10 months of age.

In these children, neurodevelopmental delay at 6 months old was observed at similar rates in boys (8.9 %) and girls (8.5 %), whereas that at 12 months old was more frequently identified in boys (16.9 %) than in girls (12.6 %). The baseline characteristics of the children who did not have all data available were comparable to those of children with complete data, but their parents tended to more often be multipara, have a smoking habit and have less education and income than those of complete-data subjects (Table 1). The comparisons between children with and without anthropometric data using other combinations among 4, 7 and 10 months old showed similar tendencies (Table S1, S2, S3).

**Table 1:** Baseline characteristics of the children with and without all anthropometric data at 4, 7 and 10 months old (*n* = 86,805)

	Boy					Girl				
	With data(*n* = 10,016)	Missing	Without data(*n* = 34,248)	Missing	Effect size^a^	With data(*n* = 9,801)	Missing	Without data(*n* = 32,740)	Missing	Effect size^a^
Gestational age, mean (SD), wk	39.4 (1.1)	0	39.4 (1.1)	0	0.00	39.5 (1.1)	0	39.5 (1.1)	0	0.00
Birth length, mean (SD), cm	49.4 (2.0)	0	49.4 (1.9)	0	0.01	48.8 (1.9)	0	48.8 (1.9)	0	0.01
Birth weight, mean (SD), g	3,088 (371)	0	3,117 (367)	0	0.03	2,993 (356)	0	3,021 (357)	0	0.03
Multipara (%)	5,179 (51.7)	0	21,291 (62.2)	0	0.09	5,040 (51.4)	0	20,455 (62.5)	0	0.09
Maternal age, mean (SD), y	31.4 (4.8)	1	31.1 (5.1)	4	0.03	31.4 (4.9)	0	31.0 (5.1)	1	0.03
Maternal smoking (%)	1,435 (14.5)	112	6,418 (19.3)	948	0.05	1,429 (14.7)	109	6,167 (19.4)	891	0.05
Maternal education (%)		83		856	0.05		88		799	0.06
Junior high school	321 (3.2)		1,798 (5.4)			282 (2.9)		1,690 (5.3)		
High school	7,233 (72.8)		24,661 (73.9)			7,082 (72.9)		23,732 (74.3)		
University/graduate school	2,379 (24.0)		6,933 (20.8)			2,349 (24.2)		6,519 (20.4)		
Paternal education (%)		137		1,073	0.04		137		1,010	0.04
Junior high school	520 (5.3)		2,619 (7.9)			545 (5.6)		2,527 (8.0)		
High school	5,887 (59.6)		19,713 (59.4)			5,748 (59.5)		18,882 (59.5)		
University/graduate school	3,472 (35.1)		10,843 (32.7)			3,371 (34.9)		10,321 (32.5)		
Family income (%)		723		3,207	0.04		687		2,882	0.03
Low	3,428 (36.9)		12,719 (41.0)			3,453 (37.9)		12,232 (41.0)		
Middle	3,162 (34.0)		10,242 (33.0)			2,998 (32.9)		9,837 (32.9)		
High	2,703 (29.1)		8,080 (26.0)			2,663 (29.2)		7,789 (26.1)		
Home speech stimulation (%)	8,115 (81.4)	50	26,998 (81.1)	950	0.00	7,916 (81.4)	75	25,879 (81.3)	901	0.00
ND at 6 months (%)	841 (8.9)	599	2,439 (8.5)	5,636	0.01	782 (8.5)	595	2,169 (7.9)	5,215	0.01
ND at 12 months (%)	1,576 (16.9)	712	4,253 (15.8)	7,324	0.01	1,221 (13.6)	819	3,247 (12.6)	6,945	0.01

^a^Effect size indicates the strength of the difference between groups and is calculated as *phi*/Cramer’s *V* and *r*, using chi-square and Student’s *t*-tests for categorical and numerical variables, respectively.

*ND* neurodevelopmental delay; *SD* standard deviation

Multivariable regression models revealed that a reduced risk of neurodevelopmental delay at 6 months old was found in boys with a higher birth weight (aRR: 0.91, 95 % CI: 0.87–0.96) and an increased conditional length at 4 months old (aRR: 0.85, 95 % CI: 0.82–0.88) (Table 2), and similarly in girls with a higher birth weight (aRR: 0.93, 95 % CI: 0.88–0.98) and an increased conditional length at 4 months old (aRR: 0.87, 95 % CI: 0.83–0.91) (Table 3). On analyzing each neurodevelopmental domain of ASQ-3, the conditional length at four months old was associated with all five domains, while the birth weight was associated with some domains, supporting the stronger effect sizes of conditional length at four months old than those of birth weight for both sexes.

**Table 2:** Association between conditional variables and neurodevelopmental delay at six months old in boys

Conditional variables at birth and 4 months old	Total	Communication	Gross motor	Fine motor	Problem solving	Personal-Social
	2,714/31,399 (8.6 %)	578/31,595 (1.8 %)	526/31,590 (1.7 %)	823/31,506 (2.6 %)	1,204/31,590 (3.8 %)	492/31,539 (1.6 %)
Length at birth	0.99 (0.95–1.04)	0.93 (0.83–1.03)	0.90 (0.80-1.00)	1.01 (0.92–1.10)	1.01 (0.94–1.09)	1.02 (0.91–1.15)
Weight at birth	**0.91 (0.87–0.96)**	1.02 (0.92–1.14)	0.97 (0.86–1.08)	**0.88 (0.80–0.96)**	**0.88 (0.82–0.95)**	**0.87 (0.77–0.98)**
cLength at 4 months old	**0.85 (0.82–0.88)**	**0.84 (0.76–0.92)**	**0.83 (0.77–0.90)**	**0.82 (0.77–0.88)**	**0.84 (0.79–0.90)**	**0.84 (0.77–0.92)**
crWeight at 4 months old	1.03 (0.98–1.08)	**0.88 (0.79–0.99)**	1.24 (1.11–1.38)	0.99 (0.90–1.09)	0.98 (0.90–1.06)	1.11 (0.98–1.25)

Data represent the adjusted relative risk (95 % confidence interval). All of the conditional variables and the following covariates were included together in the model: maternal age and smoking, maternal and paternal education, family income, and home speech stimulation.

*cLength* conditional length; *crWeight* conditional relative weight

**Table 3:** Association between conditional variables and neurodevelopmental delay at six months old in girls

Conditional variables at birth and 4 months old	Total	Communication	Gross motor	Fine motor	Problem solving	Personal-Social
	2,432/30,358 (8.0 %)	584/30,504 (1.9 %)	468/30,505 (1.5 %)	743/30,436 (2.4 %)	1,113/30,510 (3.6 %)	290/30,476 (1.0 %)
Length at birth	0.99 (0.95–1.05)	1.04 (0.93–1.16)	1.12 (0.99–1.26)	0.98 (0.89–1.07)	0.96 (0.89–1.04)	0.90 (0.78–1.05)
Weight at birth	**0.93 (0.88–0.98)**	0.93 (0.84–1.04)	**0.83 (0.73–0.94)**	0.96 (0.87–1.06)	0.95 (0.88–1.02)	0.94 (0.81–1.10)
cLength at 4 months old	**0.87 (0.83–0.91)**	**0.86 (0.78–0.94)**	**0.89 (0.81–0.99)**	**0.88 (0.81–0.96)**	**0.83 (0.78–0.89)**	**0.80 (0.72–0.91)**
crWeight at 4 months old	1.05 (0.99–1.11)	0.92 (0.82–1.03)	**1.44 (1.26–1.63)**	0.99 (0.89–1.10)	0.99 (0.91–1.07)	1.14 (0.97–1.34)

Data represent the adjusted relative risk (95 % confidence interval). All of the conditional variables and the following covariates were included together in the model: maternal age and smoking, maternal and paternal education, family income, and home speech stimulation.

*cLength* conditional length; *crWeight* conditional relative weight

With regard to neurodevelopmental delay at 12 months old, we first created a regression model using the data of children who had complete anthropometric data throughout the first year of age and all covariates (Tables 4 and 5, model 1). In boys, a reduced risk was related to an increased conditional length at 4 months old (aRR: 0.92, 95 % CI: 0.87–0.98) and an increased conditional relative weight at 4 months old (aRR: 0.90, 95 % CI: 0.84–0.97) (Table 4). In girls, a reduced risk was related to a higher birth weight (aRR: 0.89, 95 % CI: 0.83–0.96) and an increased conditional length at 4 months old (aRR: 0.90, 95 % CI: 0.84–0.96) (Table 5). Analyses of each neurodevelopmental domain revealed that, for both sexes, conditional length at four months old was most consistently associated with several domains compared with other conditional variables, again suggesting the importance of this variable. Conversely, the risk was largely unrelated to conditional variables at 7 and 10 months old, although an increased risk was paradoxically observed in girls with an increased conditional length at 7 months old (aRR: 1.16, 95 % CI: 1.07–1.26) (Table 5). We also built 2 other regression models using the data of children with available data from 4 to 7 months old (model 2) and those with available data from 4 to 10 months old (model 3). The results of the two models included some inconsistencies but yielded similar results concerning the importance of conditional variables in early infancy.

**Table 4:** Association between conditional variables and neurodevelopmental delay at 12 months old in boys

**[Model 1] Conditional variables at birth, 4, 7 and 10 months old**	**Total**	**Communication**	**Gross motor**	**Fine motor**	**Problem solving**	**Personal-Social**
	**1,441/8,488 (17.0 %)**	**396/8,534 (4.6 %)**	**458/8,532 (5.4 %)**	**372/8,526 (4.4 %)**	**511/8,519 (6.0 %)**	**303/8,506 (3.6 %)**
Length at birth	1.00 (0.94–1.07)	0.99 (0.87–1.12)	1.01 (0.90–1.13)	1.03 (0.90–1.17)	0.97 (0.87–1.09)	1.00 (0.86–1.16)
Weight at birth	0.97 (0.91–1.03)	0.97 (0.85–1.10)	0.96 (0.86–1.08)	1.00 (0.88–1.14)	0.97 (0.87–1.08)	0.92 (0.79–1.06)
cLength at 4 months old	**0.92 (0.87–0.98)**	**0.87 (0.78–0.98)**	0.98 (0.89–1.09)	**0.81 (0.73–0.91)**	**0.86 (0.78–0.95)**	**0.79 (0.70–0.90)**
crWeight at 4 months old	**0.90 (0.84–0.97)**	0.96 (0.83–1.10)	0.88 (0.77-1.00)	0.89 (0.78–1.03)	**0.86 (0.76–0.97)**	**0.84 (0.72–0.98)**
cLength at 7 months old	1.07 (1.00-1.14)	1.14 (0.99–1.30)	1.13 (0.99–1.28)	1.08 (0.94–1.24)	1.05 (0.95–1.18)	1.01 (0.89–1.18)
crWeight at 7 months old	0.98 (0.89–1.07)	0.81 (0.65-1.00)	0.91 (0.74–1.10)	0.90 (0.72–1.10)	1.00 (0.85–1.15)	1.03 (0.86–1.22)
cLength at 10 months old^a^	0.98 (0.90–1.07)	0.91 (0.77–1.08)	1.13 (0.95–1.33)	1.00 (0.83–1.20)	1.10 (0.94–1.27)	0.87 (0.73–1.05)
crWeight at 10 months old^b^	0.94 (0.87–1.03)	0.94 (0.83–1.16)	0.90 (0.81–1.05)	1.02 (0.85–1.33)	0.96 (0.84–1.17)	0.97 (0.81–1.26)
**[Model 2] Conditional variables at birth, 4 and 7 months old**	**Total**	**Communication**	**Gross motor**	**Fine motor**	**Problem solving**	**Personal-Social**
	**2,281/13,689 (16.7 %)**	**612/13,757 (4.4 %)**	**698/13,759 (5.1 %)**	**614/13,753 (4.5 %)**	**814/13,742 (5.9 %)**	**481/13,722 (3.5 %)**
Length at birth	1.02 (0.97–1.07)	1.00 (0.90–1.10)	1.03 (0.94–1.14)	1.01 (0.91–1.12)	1.00 (0.91–1.09)	1.04 (0.93–1.18)
Weight at birth	0.96 (0.91–1.01)	0.96 (0.87–1.07)	0.94 (0.85–1.03)	0.97 (0.87–1.07)	0.94 (0.86–1.03)	0.90 (0.80–1.01)
cLength at 4 months old	**0.93 (0.89–0.97)**	**0.88 (0.80–0.97)**	0.97 (0.89–1.06)	**0.85 (0.78–0.93)**	**0.89 (0.82–0.97)**	**0.81 (0.73–0.90)**
crWeight at 4 months old	**0.90 (0.85–0.95)**	**0.88 (0.79–0.99)**	**0.89 (0.80–0.99)**	0.91 (0.81–1.01)	**0.85 (0.77–0.93)**	0.90 (0.79–1.01)
cLength at 7 months old	**1.08 (1.02–1.14)**	1.09 (0.97–1.23)	**1.14 (1.02–1.27)**	**1.16 (1.03–1.29)**	**1.11 (1.00-1.23)**	1.05 (0.95–1.19)
crWeight at 7 months old	1.00 (0.92–1.08)	0.87 (0.72–1.04)	0.99 (0.84–1.15)	0.94 (0.79–1.10)	0.99 (0.86–1.11)	1.15 (0.99–1.31)
**[Model 3] Conditional variables at birth, 4 and 10 months old**	**Total**	**Communication**	**Gross motor**	**Fine motor**	**Problem solving**	**Personal-Social**
	**3,478/21,577 (16.1 %)**	**910/21,687 (4.2 %)**	**1086/21,684 (5.0 %)**	**923/21,677 (4.3 %)**	**1,232/21,656 (5.7 %)**	**723/21,626 (3.3 %)**
Length at birth	1.00 (0.96–1.04)	0.93 (0.85–1.01)	0.98 (0.91–1.06)	0.99 (0.91–1.08)	1.02 (0.95–1.10)	0.97 (0.88–1.07)
Weight at birth	0.97 (0.93–1.01)	0.98 (0.90–1.06)	0.98 (0.91–1.06)	0.99 (0.91–1.08)	0.93 (0.87-1.00)	0.92 (0.83–1.01)
cLength at 4 months old	**0.92 (0.89–0.95)**	**0.85 (0.79–0.92)**	0.97 (0.90–1.04)	**0.85 (0.79–0.91)**	**0.87 (0.82–0.93)**	**0.83 (0.76–0.90)**
crWeight at 4 months old	**0.92 (0.88–0.96)**	0.94 (0.85–1.03)	**0.91 (0.84-1.00)**	**0.89 (0.82–0.98)**	**0.86 (0.80–0.93)**	**0.87 (0.79–0.96)**
cLength at 10 months old^a^	1.00 (0.96–1.05)	**0.91 (0.85–0.99)**	1.00 (0.92–1.09)	1.00 (0.91–1.09)	1.05 (0.97–1.14)	1.01 (0.92–1.11)
crWeight at 10 months old^b^	1.02 (0.97–1.07)	1.01 (0.92–1.11)	0.91 (0.85–0.99)	0.96 (0.88–1.06)	1.01 (0.93–1.10)	1.08 (0.98–1.17)

Data represent the adjusted relative risk (95 % confidence interval). All of the conditional variables and the following covariates were included together in the model: maternal age and smoking, maternal and paternal education, family income, and home speech stimulation. ^a,b^The values of the variables were different between the models because these variables were estimated from different combinations of the previous data ([model 1] at birth, 4 and 7 months old; [model 3] at birth and 4 months old).

*cLength* conditional length; *crWeight* conditional relative weight

**Table 5:** Association between conditional variables and neurodevelopmental delay at 12 months old in girls

**[Model 1] Conditional variables at birth, 4, 7 and 10 months old**	**Total**	**Communication**	**Gross motor**	**Fine motor**	**Problem solving**	**Personal-Social**
	**1,117/8,211 (13.6 %)**	**199/8,253 (2.4 %)**	**540/8,254 (6.5 %)**	**235/8,254 (2.8 %)**	**348/8,244 (4.2 %)**	**202/8,229 (2.5 %)**
Length at birth	1.04 (0.97–1.12)	0.92 (0.77–1.11)	1.05 (0.94–1.17)	1.05 (0.88–1.24)	1.01 (0.88–1.16)	0.99 (0.82–1.18)
Weight at birth	**0.89 (0.83–0.96)**	0.96 (0.80–1.16)	**0.87 (0.78–0.97)**	0.87 (0.73–1.03)	**0.86 (0.75–0.99)**	0.94 (0.78–1.13)
cLength at 4 months old	**0.90 (0.84–0.96)**	0.85 (0.73–1.01)	1.02 (0.92–1.12)	**0.77 (0.66–0.90)**	**0.82 (0.73–0.93)**	**0.78 (0.67–0.92)**
crWeight at 4 months old	0.96 (0.89–1.04)	**0.80 (0.66–0.97)**	0.94 (0.84–1.05)	0.97 (0.81–1.16)	0.91 (0.79–1.06)	0.88 (0.72–1.06)
cLength at 7 months old	**1.16 (1.07–1.26)**	1.17 (0.95–1.43)	1.21 (1.08–1.37)	1.06 (0.91–1.29)	1.14 (0.98–1.33)	1.10 (0.91–1.35)
crWeight at 7 months old	1.10 (0.98–1.23)	0.91 (0.67–1.20)	1.19 (1.00-1.39)	0.92 (0.70–1.21)	1.01 (0.81–1.25)	0.98 (0.73–1.28)
cLength at 10 months old^a^	0.97 (0.88–1.08)	0.94 (0.73–1.21)	1.04 (0.89–1.21)	1.04 (0.82–1.32)	1.07 (0.88–1.29)	0.87 (0.69–1.12)
crWeight at 10 months old^b^	1.02 (0.91–1.18)	0.87 (0.73–1.17)	1.03 (0.87–1.27)	1.17 (0.86–1.65)	1.23 (0.94–1.63)	0.90 (0.75–1.23)
**[Model 2] Conditional variables at birth, 4 and 7 months old**	**Total**	**Communication**	**Gross motor**	**Fine motor**	**Problem solving**	**Personal-Social**
	**1,751/13,220 (13.2 %)**	**313/13,289 (2.4 %)**	**810/13,293 (6.1 %)**	**371/13,292 (2.8 %)**	**544/13,278 (4.1 %)**	**317/13,250 (2.4 %)**
Length at birth	1.01 (0.95–1.07)	0.93 (0.80–1.08)	1.06 (0.97–1.16)	1.01 (0.88–1.16)	0.94 (0.84–1.05)	0.98 (0.85–1.14)
Weight at birth	**0.94 (0.88–0.99)**	0.95 (0.82–1.10)	**0.86 (0.78–0.94)**	0.96 (0.84–1.10)	0.96 (0.86–1.07)	0.95 (0.82–1.10)
cLength at 4 months old	**0.90 (0.86–0.95)**	**0.85 (0.75–0.97)**	1.01 (0.94–1.10)	**0.83 (0.74–0.94)**	**0.82 (0.75–0.91)**	**0.81 (0.71–0.92)**
crWeight at 4 months old	**0.93 (0.88–0.99)**	**0.78 (0.67–0.92)**	0.92 (0.83–1.01)	0.96 (0.84–1.11)	**0.89 (0.79–0.99)**	0.94 (0.81–1.09)
cLength at 7 months old	**1.17 (1.10–1.26)**	1.13 (0.96–1.35)	**1.22 (1.10–1.35)**	1.15 (0.98–1.34)	**1.14 (1.01–1.30)**	1.07 (0.92–1.27)
crWeight at 7 months old	1.10 (0.99–1.21)	0.89 (0.72–1.13)	1.19 (1.03–1.37)	0.94 (0.76–1.18)	1.07 (0.89–1.27)	0.98 (0.78–1.24)
**[Model 3] Conditional variables at birth, 4 and 10 months old**	**Total**	**Communication**	**Gross motor**	**Fine motor**	**Problem solving**	**Personal-Social**
	**2,669/20,756 (12.9 %)**	**441/20,857 (2.1 %)**	**1,263/20,864 (6.1 %)**	**537/20,858 (2.6 %)**	**827/20,837 (4.0 %)**	**463/20,799 (2.2 %)**
Length at birth	1.02 (0.97–1.07)	0.95 (0.84–1.07)	1.03 (0.95–1.10)	1.03 (0.92–1.15)	1.03 (0.94–1.12)	1.04 (0.92–1.17)
Weight at birth	**0.92 (0.88–0.97)**	0.95 (0.84–1.08)	**0.92 (0.85–0.99)**	**0.89 (0.79–0.99)**	**0.91 (0.83–0.99)**	**0.87 (0.77–0.98)**
cLength at 4 months old	**0.91 (0.88–0.95)**	**0.85 (0.77–0.95)**	0.95 (0.89–1.01)	**0.88 (0.80–0.97)**	**0.86 (0.80–0.93)**	**0.87 (0.78–0.97)**
crWeight at 4 months old	0.98 (0.93–1.03)	**0.86 (0.76–0.99)**	0.98 (0.91–1.06)	0.94 (0.83–1.06)	0.96 (0.87–1.05)	0.88 (0.78–1.01)
cLength at 10 months old^a^	**1.06 (1.01–1.12)**	0.99 (0.88–1.13)	1.06 (0.98–1.15)	1.08 (0.96–1.22)	**1.15 (1.04–1.26)**	1.02 (0.90–1.16)
crWeight at 10 months old^b^	1.05 (0.99–1.13)	0.98 (0.84–1.15)	1.04 (0.94–1.14)	0.96 (0.85–1.11)	1.05 (0.93–1.18)	0.95 (0.83–1.11)

Data represent the adjusted relative risk (95 % confidence interval). All of the conditional variables and the following covariates were included together in the model: maternal age and smoking, maternal and paternal education, family income, and home speech stimulation. ^a,b^The values of the variables were different between the models because these variables were estimated from different combinations of the previous data ([model 1] at birth, 4 and 7 months old; [model 3] at birth and 4 months old).

*cLength* conditional length; *crWeight* conditional relative weight

## Discussion

The present study investigated the relationship between physical growth and neurodevelopmental delay during the first year of life in term-born infants. As indices of physical growth, we discriminated linear growth from relative weight gain, represented as ‘conditional length’ and ‘conditional relative weight’, respectively. A reduced risk of neurodevelopmental delay at six months old was observed in boys and girls with a higher birth weight and an increased conditional length at four months old. A reduced risk of neurodevelopmental delay at 12 months old was found in boys and girls with a higher conditional length at 4 months old, boys with a relatively high conditional relative weight at 4 months old and girls with a higher birth weight. These findings indicate that children with a poor growth in early infancy are at an increased risk for neurodevelopmental delay. In contrast, conditional variables at 7 and 10 months old did not increase the risk.

This was one of the largest birth cohort studies yet conducted to examine the association between physical growth and the neurodevelopmental outcome. Our large sample size had strong statistical power and revealed subtle but significant associations. Furthermore, body sizes were converted to z-scores using domestically standardized growth curves, and the sizes at birth were further adjusted for the mother’s parity and child’s gestational age, enabling the precise estimation of the body growth *in utero*. However, the present study included several limitations. First, the information was mainly collected via self-administered questionnaires. Although the anthropometric data were based on official records, the identified neurodevelopmental delay might be somewhat equivocal, relying on the parent-reported screening test of the Japanese version of ASQ-3. Next, a considerable proportion of children had missing information on physical growth at different time points and neurodevelopmental outcomes and were thus excluded from the analysis, possibly producing selection bias. Finally, the results of our regression analyses were not adjusted for other unmeasured confounders, including the maternal intelligent quotient, which greatly influences the neurocognitive development of offspring.

We found that a higher conditional length at 4 months old was consistently associated with a reduced risk of neurodevelopmental delay at 6 and 12 months of age for both sexes. The results agree with the findings from previous studies using conditional modeling, which reported a greater impact of linear growth than relative weight gain on schooling and intelligence [19, 20]. The biological mechanisms underlying this association remains unclear but is likely multifactorial. Prenatal and postnatal nutritional deficiency can induce growth failure of the body and brain, as observed in low-income countries [36]. Although severe malnutrition is less likely to occur in Japan than in other countries, placental dysfunction and maternal excessive dieting may cause a poor nutrient supply to the fetus, while inappropriate feeding habits might produce nutritional insufficiency during infancy. Another possible explanation is hormonal factors, such as the growth hormone/insulin-like growth factor-I pathway and the hypothalamic-pituitary-gonadal axis. These endocrine systems are regulated by nutritional and genetic factors [37–41] and play key roles in both the physical and cerebral growth of the fetus and child [42–46]. Subtle differences in such hormonal levels might be responsible for the association between physical growth and neurodevelopment.

We also found other associations between physical growth and neurodevelopmental delay during infancy. The association between a higher birth weight and a reduced risk of neurodevelopmental delay at 6 and 12 months old is concordant with the well-known finding that birth weight is a significant determinant of later neurodevelopment [1, 47–49]. The boy-specific association between the conditional relative weight at 4 months and neurodevelopmental delay at 12 months was in line with the association between conditional relative weight and school achievement reported by Adair et al. [20]. The aforementioned endocrine factors have differential effects on somatic and cognitive development between sexes [46, 50] and might account at least partially for this association. Conversely, higher conditional variables at 7 and 10 months old did not reduce the risk of neurodevelopmental delay at 12 months, suggesting less susceptibility of the developing brain in late infancy than in early infancy. An increased risk at 12 months was also paradoxically observed in girls with an increased conditional length at either 7 or 10 months old in all the regression models. When an infant shows slow growth in early infancy, they may be inclined to be fed more by their parents and thus may grow faster thereafter. Such modification of parents’ feeding behavior might yield this paradoxical association, although this information was not collected in the JECS.

## Conclusions

Using appropriate conditional modeling, we showed that poor early physical growth was associated with an increased risk of neurodevelopment delay during the first year of life in term-born infants partly in a sex-dependent manner. If a child presents with growth failure in early infancy, the infant should be monitored carefully to detect neurodevelopmental delay for early intervention. Ongoing investigations in the JECS cohort may reveal later-term outcomes.

## Supplementary Information

**Additional file 1:**

## Data Availability

Data are unsuitable for public deposition due to ethical restrictions and the legal framework of Japan. It is prohibited by the Act on the Protection of Personal Information (Act No. 57 of 30 May 2003, amendment on 9 September 2015) to publicly deposit data containing personal information. The Ethical Guidelines for Medical and Health Research Involving Human Subjects enforced by the Japan Ministry of Education, Culture, Sports, Science and Technology and the Ministry of Health, Labour and Welfare also restricts the open sharing of the epidemiologic data. All inquiries about access to data should be sent to: jecs-en@nies.go.jp. The person responsible for handling enquiries sent to this e-mail address is Dr Shoji F. Nakayama, JECS Programme Office, National Institute for Environmental Studies.

## Abbreviations

aRR: Adjusted relative risk
ASQ-3: Ages & Stages Questionnaires, third edition
CI: Confidence interval
JECS: The Japan Environment and Children’s Study
JPY: Japanese Yen
